# Domain-Adaptive Artificial Intelligence-Based Model for Personalized Diagnosis of Trivial Lesions Related to COVID-19 in Chest Computed Tomography Scans

**DOI:** 10.3390/jpm11101008

**Published:** 2021-10-07

**Authors:** Muhammad Owais, Na Rae Baek, Kang Ryoung Park

**Affiliations:** Division of Electronics and Electrical Engineering, Dongguk University, 30 Pildong-ro 1-gil, Jung-gu, Seoul 04620, Korea; malikowais266@gmail.com (M.O.); naris27@dgu.ac.kr (N.R.B.)

**Keywords:** artificial intelligence, COVID-19 infection segmentation, computer-aided diagnosis, lung disease, DAL-Net

## Abstract

Background: Early and accurate detection of COVID-19-related findings (such as well-aerated regions, ground-glass opacity, crazy paving and linear opacities, and consolidation in lung computed tomography (CT) scan) is crucial for preventive measures and treatment. However, the visual assessment of lung CT scans is a time-consuming process particularly in case of trivial lesions and requires medical specialists. Method: A recent breakthrough in deep learning methods has boosted the diagnostic capability of computer-aided diagnosis (CAD) systems and further aided health professionals in making effective diagnostic decisions. In this study, we propose a domain-adaptive CAD framework, namely the dilated aggregation-based lightweight network (DAL-Net), for effective recognition of trivial COVID-19 lesions in CT scans. Our network design achieves a fast execution speed (inference time is 43 ms on a single image) with optimal memory consumption (almost 9 MB). To evaluate the performances of the proposed and state-of-the-art models, we considered two publicly accessible datasets, namely COVID-19-CT-Seg (comprising a total of 3520 images of 20 different patients) and MosMed (including a total of 2049 images of 50 different patients). Results: Our method exhibits average area under the curve (AUC) up to 98.84%, 98.47%, and 95.51% for COVID-19-CT-Seg, MosMed, and cross-dataset, respectively, and outperforms various state-of-the-art methods. Conclusions: These results demonstrate that deep learning-based models are an effective tool for building a robust CAD solution based on CT data in response to present disaster of COVID-19.

## 1. Introduction

The highly infectious coronavirus disease 2019 (COVID-19) has distressed millions of people worldwide. Based on the statistics of the World Health Organization (WHO) [1] on March 29, 2021, approximately 126,359,540 confirmed COVID-19 cases, including 2,769,473 deaths, have been reported worldwide. Recently, a new variant of COVID-19 has further threatened the whole world because of its more transmissible impact and nature. In the context of COVID-19 treatment, a few vaccines [2] have completed rigorous clinical trials and acquired the Food and Drug Administration (FDA) approval. However, the vast production and global distribution of COVID-19 vaccines remain a challenging and time-consuming task. Early and effective diagnosis of this infection is a crucial and key preventive measure to overcome its worldwide commutability. Currently, molecular tests, such as reverse transcription-polymerase chain reaction (RT-PCR), are being carried out for the clinical diagnosis of positive cases [3]. Nevertheless, subjective assessment with strict clinical requirements may constrain the usability of such diagnostic methods in a real-time screening environment. 

In recent studies [3,4], chest computed tomography (CT) scans have been recognized as cost-effective diagnostic measures for the visual detection of COVID-19. The experimental results reported in [3] indicated that the visual assessment of CT images achieved a sensitivity of 97% compared to the RT-PCR results. Similar findings in [4,5] revealed the effectiveness of radiological imaging modalities in the early assessment of COVID-19 virus. However, the personal assessment of chest CT scans is also time-taking process particularly in case of trivial lesions and requires medical professionals. Recently, the advancements in artificial intelligence (AI) technology, particularly related to medical diagnostic domains [6,7,8,9,10,11,12,13,14,15,16,17,18,19,20], have replaced various subjective diagnostic methods with efficient computer-aided diagnosis (CAD) solutions. Generally, a CAD tool analyzes the given image using a set of AI algorithms and ultimately makes a diagnostic decision. Recently, a new set of AI algorithms, known as deep learning, has substantially improved the diagnostic capability of various CAD methods. Such advanced deep learning algorithms can simulate the human brain’s power to achieve diagnostic results comparable to those of medical experts. Recently, convolutional neural networks (CNNs), a well-known variant of deep learning algorithms, have gained special attention in the context of image-based diagnosis. The internal layout of a CNN model is mainly composed of a set of convolutional followed by fully connected (FC) layers, including some training parameters. These parameters are responsible for exploiting the key features from the given data sample and are initially trained using an independent training dataset. After sufficient training, a deep CNN model can analyze the testing data sample and generate the desired output.

In the literature [8,9,10,11,12,13,14,15,16,17,18,19,20], different types of CNN-based CAD tools have been proposed for the diagnosis of COVID-19 virus using chest radiographic images. For example, Oh et al. [8], Owais et al. [9], and Lee et al. [10] proposed classification-based CAD solutions by providing novel training schemes to perform sufficient training of a deep network in the context of limited data samples. However, these models [8,9,10] were trained to perform the classification of COVID-19-positive and -negative cases only. Semantic segmentation models perform well in localizing the lesions of COVID-19 infection in a given X-ray or CT image. However, well-annotated segmentation masks are required to perform sufficient training and validation of these segmentation models. Such data annotation is time-consuming and requires medical experts. To overcome the requirement of large-scale annotated data related to COVID-19, data synthesis [11,12] and semi-supervised learning [13] methods have been proposed to efficiently train a deep network.

To accelerate the development of data-efficient deep networks for the effective diagnosis of COVID-19, Ma et al. [14] developed three benchmarks for both lung and virus segmentation. In a recent study, Oulefki et al. [15] utilized conventional machine learning techniques in COVID-19 diagnosis using limited annotated data samples. Later, Abdel-Basset et al. [18] proposed a data efficient segmentation model to overcome the requirement of large-scale annotated data. Subsequently, El-Bana et al. [16] proposed a multi-task classification and segmentation pipeline using existing CNN models, namely Inception-v3 [21] and DeepLabV3+ [22]. Later, Selvaraj et al. [19] proposed another classification and segmentation pipeline using a combination of deep and handcrafted features. To deal with the small lesion segmentation of COVID-19 infection, Zheng et al. [17] proposed a multi-scale discriminative network (MSD-Net) with the ability to detect infected areas of various sizes. Subsequently, Zhou et al. [20] presented a modified U-Net architecture using an attention mechanism with the capability of capturing deep contextual relationships. 

Most of the existing studies [11,13,15,16,17,18,19,20] did not perform cross-data analysis to highlight the generality of their methods. A few studies [12,14] based on cross-data analysis do exist; however, their results are significantly lower than those of our method. Consequently, to address the limitations of existing methods, a lightweight segmentation model is proposed that outperforms various existing methods in terms of both quantitative and computational measures. The major contributions of our method are as follows.

We proposed a dilated aggregation-based lightweight network (DAL-Net) for COVID-19 diagnosis in chest CT scans (with a total of 6.65 million parameters), which utilizes the strength of efficient depth-wise (DW) convolution and dilated (DL) convolution, resulting in a fast execution speed (average inference time of 43 ms on a single image) and optimal memory consumption (almost 9 MB).

We used the atrous block (A-Block) in the residual connection to incorporate multi-scale contextual features with high-frequency information, which ultimately resulted in better performance, particularly in the case of small lesions.

To highlight the generality of the proposed DAL-Net in a real-world scenario, we also performed cross-data analysis and further enhanced its generalization capability by applying the Reinhard (RH) transformation [23].

Our proposed DAL-Net attained a new state-of-the-art performance on the COVID-19-CT-Seg [14,24] and MosMed [25] datasets. In addition, we evaluated the performance of various state-of-the-art deep segmentation networks to provide standard benchmarks, particularly in the context of a cross-dataset setting.

Finally, we rendered our DAL-Net publicly available for research and educational purposes through [26].

The rest of this paper is arranged as follows: Section 2 describes the selected datasets and proposed method with a focus on the network structure and workflow. Section 3 explains the experimental setting and quantitative results. Finally, a brief discussion and conclusion are given in Section 4 and Section 5, respectively.

## 2. Material and Methods

### 2.1. Datasets

**Figure 1:**
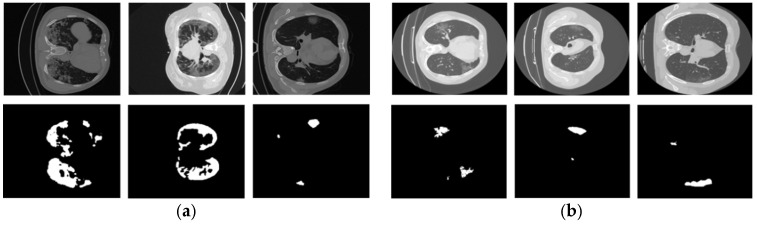
Example data samples of COVID-19-positive cases as voxel images and their corresponding ground-truth masks of (**a**) COVID-19-CT-Seg data and (**b**) MosMed data.

Our proposed DAL-Net was validated using two publicly accessible CT datasets: COVID-19-CT-Seg [14,24] and MosMed [25]. Figure 1 shows a few examples of positive data samples as voxel images and their corresponding annotations as binary masks for both datasets. The COVID-19-CT-Seg dataset comprises 20 COVID-19-positive cases (proportion of infected lung: 0.01% to 59%, and total voxel images: 3520) along with voxel-level annotations of the right lung, left lung, and infected regions as binary masks. In this dataset, the proportion of infected lung ranges from 0.01% to 59%. Except for these, other information, including the type of patients, country of origin or hospital, etc., is not provided in this database. All the annotations were accomplished by junior annotators. Initially, the annotation decision of each junior annotator was combined to get an average response in terms of a single segmentation mask corresponding to each CT image. Then, all the annotated masks were further improved by two medical specialists with 5 years of experience each. Finally, a senior medical specialist having more than 10 years of experience verified all of these annotations. 

The MosMed dataset includes a total of 50 COVID-19-positive cases (males: 42%, females: 56%, other/unknown: 2%, age from 18 to 97 years, median: 47 years, proportion of infected lung: ≤25%, and total voxel images: 2049) provided by municipal hospitals in Moscow, Russia. This dataset includes anonymized human lung CT scans with COVID-19-related findings. All these cases were annotated by experts in research and practical clinical centers for diagnostics and telemedicine technologies of the Mosco Health Care Department. During the annotation process of both datasets, COVID-19-related findings (such as regions of consolidation, ground-glass opacifications, bilateral, and peripheral disease) were chosen as white pixels on the binary mask of the corresponding voxel image. Except for these, other information is not provided in this database.

Both datasets are freely available for research and educational purposes through [14,24,25], and we confirm that there is no ethical issue for the present studies and using these databases.

### 2.2. Method

The main objective of the proposed segmentation model is fast execution speed and optimal memory utilization at a minimum cost in terms of performance degradation. To meet these objectives, we mainly utilize the power of DW and DL convolution in our network design and develop a lightweight network that includes a total of 6.65 million training parameters. Generally, a traditional convolutional layer [27] converts an input feature map $F_{i}$ of size $w_{i}\times h_{i}\times d_{i}$ into an output feature map $F_{j}$ of size $w_{i}\times h_{i}\times d_{j}$ by applying a filter $w\in R^{k\times k\times d_{i}\times d_{j}}$ of size k×k. This operation requires a total computational cost of $w_{i}\times h_{i}\times d_{i}\times d_{j}\times k\times k$ [27]. In contrast, a DW-convolutional layer performs a similar operation at the cost of $w_{i}\times h_{i}\times d_{i}\left(k^{2}+d_{j}\right)$ and saves the average computation cost by a factor of $k^{2}$ compared with the traditional convolution operation. Our network design mainly includes 3 × 3 DW-convolutional layers $\left(k=3\right)$, which require a total computational cost that is 8–9 times less than that of the traditional convolutional layer. In addition, the use of DL-convolution (in A-Block) results in an additional performance gain without significantly increasing the total number of parameters. A DL-convolutional layer allows the exponential expansion of the receptive field to capture the multi-scale features without influencing the computation and memory costs [22].

#### 2.2.1. Overview of the Proposed Method

**Figure 2:**
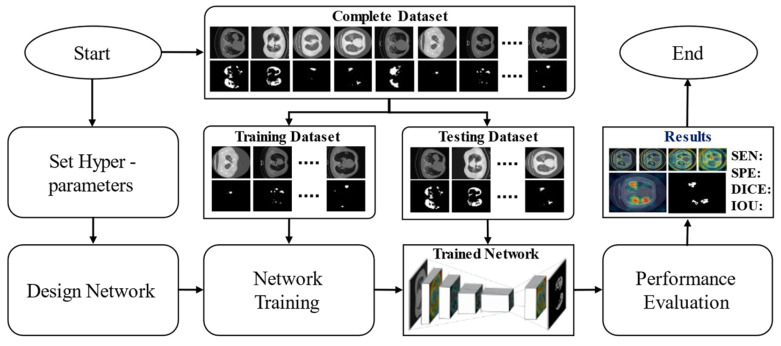
Overall workflow diagram of the proposed AI-driven CAD framework including both training and testing phases.

After selecting appropriate datasets related to COVID-19 infection, we developed a deep segmentation model intended to recognize and segment the infected regions in the given chest CT image. Initially, the proposed DAL-Net was trained for the target domain using an independent training dataset. After training, an independent testing dataset was used to assess the overall performance of the proposed model. A brief workflow (including both the training and testing phases) of the proposed AI-driven CAD framework is shown in Figure 2. In detail, our trained model performs semantic segmentation on the input CT image and classifies each pixel as either an “infection class” (pixels belong to infectious regions in the image) or “normal/background class” (pixels belonging to normal lung regions or background in the image). Thus, a binary image with a value of “1” (for infection class) and “0” (for normal/background class) is obtained as a final output of our network. 

#### 2.2.2. Network Design

The complete network design and layer-wise composition of the proposed DAL-Net are presented in Figure S1 and Table S1, respectively (Supplementary Materials). The network architecture comprises two main modules labeled as the encoder and decoder, as shown in Figure S1. Generally, the encoder module downsamples the input image to exploit the deep features, whereas the decoder upsamples the encoded image (encoder output) back to its original dimensions and generates a segmentation mask as the final output. A detailed explanation of our network design and workflow is provided in the subsequent subsections.

A. Preprocessing by Reinhard Transformation

In a real-world scenario, the testing data samples can show high intra-class variations (in color and contrast) owing to the different variants of a particular imaging modality. Generally, the generality of an AI-driven CAD tool is severely affected by high intra-class variations in the color and contrast of the given data. Therefore, a high-performance CAD model trained with only single-source data can show significant performance degradation in a real-world setting. In this study, we selected two different COVID-19 datasets with high intra-class variations and performed a cross-data (i.e., training with one dataset and testing with another dataset) analysis. We observed significant performance degradation in the case of cross-data analysis. To overcome such intra-class variations and enhance the performance of our network (in a real-world setting), we consider a simple RH transformation proposed by Reinhard et al. [23] as a pre-processing step, as shown in Figure S1 (on the left side). In the testing phase, RH transformation matches the color and contrast distribution of a validation/testing data sample to that of a training data sample by using a linear transform in a perceptual color space [28]. Mathematically, RH transforms the given testing image F into another image $F^{\prime }$ through the transformation $F^{\prime }=\tau \left(F,\varphi \right)$, where φ is a set of parameters that incorporate the visual information of a training data sample and $\tau \left(\cdot \right)$ is the RH mapping function that matches the visual appearance of the validation/testing data sample to the training data sample [23]. Finally, we obtain a normalized testing image that has a visual appearance analogous to that of the training data samples. 

B. Encoder Structure and Workflow

We designed an optimized encoder by employing basic structural units of MobileNetV2 [29] (labeled as S-Block and R-Block in Figure S1) along with a set of four multiscale DL-convolutional layers [22] (labeled as A-Block in Figure S1). Our backbone network includes a reduced number of training parameters (1.86 million), which ultimately results in a faster execution speed and lower inference time. In detail, the structure of our backbone network mainly comprises a total of 12 basic units including six stride blocks (S-Blocks), five residual blocks (R-Blocks), and one A-Block, as shown in Figure S1. Some additional convolutional layers labeled as DW-conv, conv, and pointwise (PW)-conv layers are also present as shown in Figure S1. The S-Block and R-Block (as shown in Figure S1 at the bottom left corner) comprise the following three layers: 1) a PW-convolutional layer (expansion layer): expands the depth size of the input feature map by a factor of 6; 2) a 3 × 3 DW-convolutional layer: exploits more rich features from the input feature map; and 3) a 1 × 1 PW-convolutional layer (projection layer), which decreases the depth size of the input feature map by a factor of 6. The addition of residual connection in R-Block makes it different from S-Block and overcomes the vanishing gradient problem. Whereas the S-Block mainly downsamples the input tensor using a stride of 2. Mathematically, these blocks perform the following computations:

(1) $$\psi_{S-Block}\left(F_{i},w\right)=conv\left(conv\left(conv\left(F_{i},w_{i}^{1}\right)\right),w_{i}^{2}\right),w_{i}^{3}\;$$

(2) $$\psi_{R-Block}\left(F_{i},w\right)=conv\left(conv\left(conv\left(F_{i},w_{i}^{1}\right)\right),w_{i}^{2}\right),w_{i}^{3}+F_{i}\;$$

where $\psi_{S-Block}\left(\cdot \right)$ and $\psi_{R-Block}\left(\cdot \right)$ represent the transfer functions of the S-Block and R-Block, respectively. $conv\left(\cdot \right)$ represents the convolution operation. Whereas $w_{i}^{1}$, $w_{i}^{2}$, and $w_{i}^{3}$ are the learnable parameters of expansion, the 3 × 3 DW-convolutional, and the projection layer in the *ith* S-Block and R-Block, respectively. Additionally, in both S-Block and R-Block, each convolutional layer is followed by batch normalization (BN) and an activation function called the clipped rectified linear unit (ReLU) layer. Finally, an input feature map $F_{i}^{w_{i}\times h_{i}\times d_{i}}$ of size $w_{i}\times h_{i}\times d_{i}$ undergoes the following spatial transformations after passing through these blocks: $F_{i}^{w_{i}\times h_{i}\times 6d_{i}}\to F_{i}^{w_{i}/2\times h_{i}/2\times 6d_{i}}\to F_{i}^{w_{i}/2\times h_{i}/2\times d_{i}}$ (in S-Block with a stride of 2) and $F_{i}^{w_{i}\times h_{i}\times 6d_{i}}\to F_{i}^{w_{i}\times h_{i}\times 6d_{i}}\to F_{i}^{w_{i}\times h_{i}\times d_{i}}$ (in R-Block) [9]. 

The A-Block (known as atrous spatial pyramid pooling [22], as shown in Figure S1, bottom right corner) mainly includes a total of four parallel DL-convolutional layers with different dilation rates (DR: 1, 6, 12, and 18) and effectively captures multi-scale information. For efficient computation, each DL-convolutional layer is further followed by a PW-convolutional layer (projection layer) that decreases the depth of each output feature map from 320 to 256 channels. Mathematically, the A-Block performs the following computations:

(3) $$\psi_{A-Block}\left(F_{i},w\right)=conv^{*}\left(F_{i},w_{i}^{1}\right)\;\circ \;conv^{*}\left(F_{i},w_{i}^{2}\right)\;\circ \;conv^{*}\left(F_{i},w_{i}^{3}\right)\;\circ \;conv^{*}\left(F_{i},w_{i}^{4}\right)$$

where $\psi_{A-Block}\left(\cdot \right)$ represents the transfer function of the A-Block, and $conv^{*}\left(\cdot \right)$ is the DL convolution operation. The symbol ∘ denotes the depth-concatenation operation. Mathematically, in the case of two-dimensional signals, for each particular location i,j on the input feature map $F_{i}$ and a convolution filter $w_{i}^{j}$, DL convolution is applied as follows:

(4) $$conv^{*}\left(F_{i},w_{i}^{j}\right)=\underset{l}{\sum }\underset{k}{\sum }F_{i}\left[i+r\times k,j+r\times l\right]\times w_{i}^{j}\left[k,l\right]\;$$

where the dilation rate r determines the stride with which we sample the input feature map and for r=1, DL convolution, $conv^{*}\left(\cdot \right)$, becomes the standard convolution, $conv\left(\cdot \right)$. We refer interested readers to [22] for more details.

To exploit the high-level features, the input CT image goes through a stack of different building blocks (S-Blocks, R-Blocks, and A-Block) and some additional layers [9], as shown in Figure S1. Initially, a standard convolutional layer (including a total of 32 filters of size 3 × 3) followed by a DW-convolutional layer (including a total of 32 filters of size 3 × 3) explore the image $F^{\prime }$ and produce the output tensor of size 144 × 176 × 32. Subsequently, a PW-convolutional layer (including a total of 16 filters of size 1 × 1) further explores the output of the preceding layer (i.e., output tensor of size 144 × 176 × 32) and converts it into another output tensor of size 144 × 176 × 16. After these three layers, a stack of 11 building blocks (labeled as S-Blocks 1, 2,…,6 and R-Blocks 1,2,…,5 in Figure S1) further explores high-level features. These blocks explore the output tensor of the preceding block or layer sequentially, and ultimately, we obtain an output tensor of size 18 × 18 × 320 from the last block (labeled as S-Block 6 in Figure S1). Additionally, A-Block 1 further refines the final output of S-Block 6 at multiple scales by applying a total of four parallel DL-convolutional layers with different dilation rates (DR: 1, 6, 12, and 18) and captures more diversified multi-scale features. To decrease the total number of output channels of A-Block 1, a PW-convolutional layer projects the output of A-Block 1 from 320 to 256 channels. Finally, the encoder output feature map of size 18 × 18 × 256 contains rich semantic information.

C. Decoder Structure and Workflow

We consider a simple yet effective decoder module, as illustrated in Figure S1. The decoder module comprises two transposed (TP)-convolutional layers, an A-Block (labeled as A-Block 2 in Figure S1), a SoftMax layer, and a pixel classification layer with some additional PW- and DW-convolutional layers, as shown in Figure S1. Our newly included A-Block provides a residual connection (from the encoder to the decoder) that aggregates intermediate-level multi-scale features in the decoded output of the first TP-convolutional layer. In addition, we added two additional PW-convolutional layers (before and after A-Block 2) to expand and compress the input and output features of A-Block 2. The first expansion layer increases the depth of the residual features (extracted from S-Block 2 of the encoder module) and passes the expanded output to A-Block 2, which effectively captures multi-scale information. The output of A-Block 2 contains many channels (e.g., 1024), which may outweigh the importance of the high-level encoder features (only 256 channels in our model) and make the training more difficult. Therefore, the second PW-convolutional layer projects the output of A-Block 2 from 1024 to 48 channels. Experimental results show that the conjunction of intermediate-level residual information (in the decoder module) results in an additional performance gain, particularly in the case of small lesions, at a minimal computational cost.

Initially, the encoder features of size 18 × 22 × 256 (encoder final output feature map) are bilinearly upsampled by a factor of four using the first TP-convolutional layer (including a total of 256 filters of size 8 × 8) and transformed into a new feature map of size 72 × 88 × 256. Eventually, a depth concatenation layer incorporates the multi-scale residual features of size 72 × 88 × 48 (extracted from S-Block 2 in the encoder module and further refined by A-Block 2) with the output of the first TP-convolutional layer and generates an output feature map of 72 × 88 × 304. Subsequently, a stack of five convolutional layers (including a total of two DW-convolutional layers of size 3 × 3 and three PW-convolutional layers, as explained in the decoder part of Table S1) further refined the preceding feature map (output of depth concatenation layer) and generated a new intermediate tensor of size 72 × 88 × 2. Eventually, a second TP-convolutional layer further upsamples this intermediate feature map by a factor of four and generates a final feature map of size 288 × 352 × 2. Next, the output of the TP-convolutional layer is provided to the pixel classification block comprising a SoftMax and pixel classification layer. The SoftMax layer transforms the input feature map M in terms of the probability feature map $M^{\prime }$ by applying the softmax function as $M_{i}^{\prime }=e^{M_{i}}/\sum_{i=1}^{2}e^{M_{i}}$ [27]. Later, the pixel classification layer assigns the categorical label (either “infection class” or “normal/background class”) to each feature value in the probability feature map $M^{\prime }$ (output of the SoftMax layer). Finally, a binary output image with a value of “1” (for infection class) and “0” (for normal/background class) is obtained as a final output of our network.

D. Proposed DAL-Net (Encoder) Versus Original MobileNetV2

**Figure 3:**
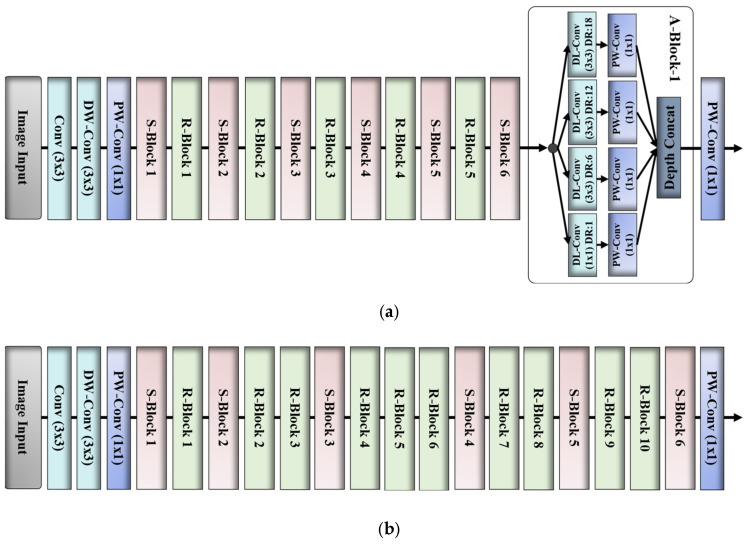
Structural difference of (**a**) our proposed encoder versus (**b**) original MobileNetV2 as the backbone network.

Our encoder design mainly comprises a total of 12 basic units including 6 S-Blocks, 5 R-Blocks, and one A-Block, whereas standard MobileNetV2 comprises a total of 16 basic units including 6 S-Blocks and 10 R-Blocks as shown in Figure 3. 

Our encoder design includes a reduced number of training parameters (specifically, 1.86 million) compared to standard MobileNetV2 (specifically, 2.24 million), which ultimately results in a faster execution speed than the MobileNetV2.

The addition of four multiscale DL-convolutional layers (labeled as A-Block-1 in Figure 3) results in an additional performance gain compared to MobileNetV2 without influencing the computation and memory costs.

#### 2.2.3. Loss Function and Network Training

Loss functions are used to calculate the deviation between predicted and actual (ground-truth) output during the training of a deep CNN model. Different types of loss functions have been presented in the literature [30]. In most of the existing studies [31,32,33], we find weighted cross-entropy loss more advantageous than simple cross-entropy loss particularly for the detection of small lesions. Therefore, we selected a weighted cross-entropy loss for the optimal training of the proposed network. Experimental results (in a later section) proved the superior performance of our selected loss function for the detection of small lesions over the simple cross-entropy loss function. In addition, instead of starting the training of our backbone model from scratch, we took advantage of the transfer learning approach [34] to build a well-trained model in a timely way. Our encoder network incudes the basic structural units (S-Block and R-Block) of MobileNetV2 along with a set of four multiscale DL-convolutional layers (A-Block). Thus, we obtain the initial weights of each S-Block and R-Block of our backbone network from the corresponding building blocks of the pre-trained MobileNetV2 encoder that was trained on a large ImageNet dataset [35] utilizing the cross-entropy loss function [27]. Therefore, we selected a relevant variant of cross-entropy loss (named as weighted cross-entropy loss) to perform the appropriate fine-tuning of our model for the target domain using a stochastic gradient descent (SGD) optimizing scheme [36]. Mathematically, the weighted cross-entropy loss function can be given as follows:

(5) $$Loss=-\frac{1}{p}\overset{p}{\underset{i=1}{\sum }}\left[\begin{array}{c} \frac{1}{\beta }\times M_{i}\times \log\left(\psi \left(F_{i},w\right)\right)+\left(\frac{1}{1-\beta }\right)\times \\ \left(1-M_{i}\right)\times \log\left(1-\psi \left(F_{i},w\right)\right) \end{array}\right]$$

where $F_{i}$ and $M_{i}$ are the $i^{th}$ training data sample and its corresponding ground-truth mask, respectively. In addition, $\psi \left(.\right)$, p, and β represent the transfer functions of our model, the total number of training samples, and the pixel frequency of the “infection class” (white pixels) in the ground-truth masks, respectively. Finally, w denotes the learnable parameters of the model. 

## 3. Results

In this section, we present the experimental setup, and results of the proposed method, along with an ablation study and comparison with state-of-the-art methods. 

### 3.1. Experimental Setup

Our proposed segmentation model was implemented in the MATLAB R2020b (MathWorks, Inc., Natick, MA, USA) coding framework using a stand-alone desktop computer with the following specifications: Intel Corei7 CPU, 16 GB RAM, NVIDIA GeForce GPU (GTX 1070), and Windows 10 operating system. In our selected optimization scheme, we used the SGD optimizer with a small learning rate value of 0.001, as used in most of the existing studies [37,38,39,40,41]. Generally, with a small learning rate, the minimum may eventually be approached; nonetheless, it will take many epochs to get there [42]. However, with a relatively large learning rate, the training loss drops rapidly at first, it fluctuates above the minimum, and never decreases to the minimum [42]. Therefore, we selected a small learning rate value for our defined optimal convergence criterion. Moreover, the following default hyperparameter settings (provided by MATLAB R2020b) were used in training: total number of epochs = 20, mini-batch size = 10, learning rate drop factor = 0.1, L2-regularization = 0.0001, and momentum factor = 0.9. In addition, we present the overall workflow of the training procedure of the proposed segmentation network as a pseudo-code in Algorithm 1.
**Algorithm 1:** Training procedure of the proposed DAL-Net**Input:**${\left\{F_{i}\right\}}_{i=1}^{p},{\left\{M_{i}\right\}}_{i=1}^{p}$: a total of p training data samples, $F_{i}$: input image, $M_{i}$: corresponding ground-truth mask **Output:** Learned parameters, $w^{\prime }$ **Parameters:** Learnable parameters, w; initial learning rate, α; maximum epoch, N; mini-batch size, B;1**Initialize parameters**w (Pre-trained weights of MobileNetV2 model that was trained on large ImageNet dataset)2**//**Continue the training procedure3**for**n=1,2,3,…,N**do  //**Loop for number of epochs 4Randomly divide the whole dataset into p/B mini-batches of size B: 
     
$\langle {\left\{F_{i}\right\}}_{i=1}^{B_{1}},{\left\{M_{i}\right\}}_{i=1}^{B_{1}}\rangle ,\langle {\left\{F_{i}\right\}}_{i=1}^{B_{2}},{\left\{M_{i}\right\}}_{i=1}^{B_{2}}\rangle ,\ldots ,\langle {\left\{F_{i}\right\}}_{i=1}^{B_{p/B}},{\left\{M_{i}\right\}}_{i=1}^{B_{p/B}}\rangle$

5** for**k=1,2,3,…,p/B**do //**Loop for number of iterations6**  obtain:**${\left\{M_{i}^{\prime }\right\}}_{i=1}^{B_{k}}=\psi \left({\left\{F_{i}\right\}}_{i=1}^{B_{k}},w\right)$/**/**$\psi \left(.\right)$ presents our model7**  update:**$w=w-\alpha .\nabla Loss\left({\left\{M_{i}^{’}\right\}}_{i=1}^{B_{k}},{\left\{M_{i}\right\}}_{i=1}^{B_{k}}\right)$/**/**loss function Equation (5) 8** end**9**end**10**//**Training stop and finally we obtain learned weights, $w^{\prime }$

To highlight the generalization capability of our model, we considered different patient data for training and testing. In our first experiment (later denoted as Exp#1), we used 80% (16/20) of the COVID-19-CT-Seg data for training and the remaining 20% (4/20) for testing. In our second experiment (later denoted as Exp#2), we considered 80% (40/50) of the MosMed data for training and the remaining 20% (10/50) for testing. For fair performance analysis, we performed five-fold cross-validation in these two experiments (Exp#1 and 2). In our third experiment (hereafter denoted as Ex#3), we performed cross-data analysis using the COVID-19-CT-Seg data as training and MosMed data as testing, and vice versa. In Exp#3, five-fold cross-validation was not viable; therefore, we performed a cross-data validation. Finally, in the testing phase, the following performance evaluation metrics were selected to evaluate the quantitative results of the proposed and other baseline models: (1) sensitivity (SEN), (2) specificity (SPE), (3) positive predictive value, (4) mean dice index (DICE), (5) mean intersection over union (IOU), and (6) area under the curve (AUC) [43]. Mathematically, these metrics are calculated as follows:

(6) $$\mathrm{SEN}=\frac{\#\mathrm{TP}}{\#\mathrm{TP}+\#\mathrm{FN}}\;$$

(7) $$\mathrm{SPE}=\frac{\#\mathrm{TN}}{\#\mathrm{TN}+\#\mathrm{FP}}\;$$

(8) $$\mathrm{PPV}=\frac{\#\mathrm{TP}}{\#\mathrm{TP}+\#\mathrm{FP}}\;$$

(9) $$\mathrm{DICE}=\frac{2\times \left(X\cap Y\right)}{X+Y}\;$$

(10) $$\mathrm{IOU}=\frac{X\cap Y}{X\cup Y}\;$$

where #TP, #TN, #FP, and #FN correspond to the numbers of true positives, true negatives, false positives, and false negatives, respectively. X and Y represent the ground-truth mask and model-predicted output, respectively.

### 3.2. Results

Table 1 presents all the quantitative results of five-fold cross-validation (in the case of the COVID-19-CT-Seg and MosMed datasets) and two-fold cross-validation (in case of the cross-dataset with and without RH transformation) based on our proposed network. The COVID-19-CT-Seg data (Exp#1) provides an average performance of 91.19%, 99.18%, 76.69%, 83.23%, 74.86%, and 98.84% for SEN, SPE, PPV, DICE, IOU, and AUC, respectively. In the case of the MosMed data (Exp#2), we obtained average performances of 89.45%, 99.41%, 62.00%, 68.63%, 61.35%, and 98.47% for SEN, SPE, PPV, DICE, IOU, and AUC, respectively. The average performance of the MosMed data was lower than that of the COVID-19-CT-Seg data. Such performance degradation (in the case of the MosMed data) results from the presence of minor lesion regions in most of the data samples. The COVID-19-CT-Seg data includes many data samples that encompass large lesion regions. In Exp#3, cross-data analysis showed significantly poor performance (i.e., 54.8%, 99.58%, 67.02%, 69.6%, 61.97%, and 87.46% for SEN, SPE, PPV, DICE, IOU, and AUC, respectively) without applying data preprocessing.

**Figure 4:**
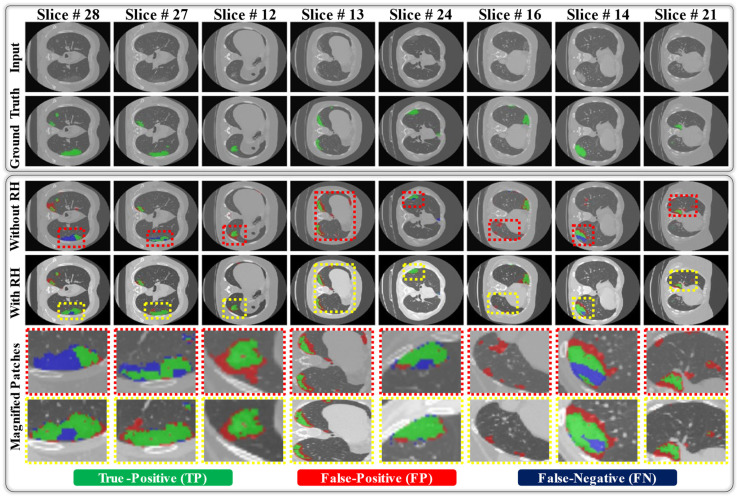
Visual output different with and without applying RH transformation in the case of cross-data analysis (Exp#3).

Based on these poor results, we further investigated the significance of RH transformation in the cross-data analysis (Exp. #3). Accordingly, we randomly selected a representative image from the training dataset as the reference image and the extracted visual information as the mapping parameters φ (as explained in [23]). Subsequently, a mapping function (as explained in [23]) was applied that transforms the visual appearance of the testing data sample to one of the training data samples using mapping parameters φ. After preprocessing all the testing data samples by applying RH transformation, we analyzed the performance of the cross-dataset (Exp#3) with the same network and achieved average gains of 18.4% [73.2%−54.8%], 2.32% [69.34%−67.02%], 5.33% [74.93%−69.6%], 4.53% [66.5%−61.97%], and 8.05% [95.51%−87.46%] for SEN, PPV, DICE, IOU, and AUC, respectively (Table 1). In addition, Figure 4 shows the visual output difference with and without the RH transformation. It can be observed that the RH transformation significantly condenses the number of FP and/or FN pixels and increases the number of TP pixels in each data sample, which ultimately results in better segmentation performance. 

**Figure 5:**
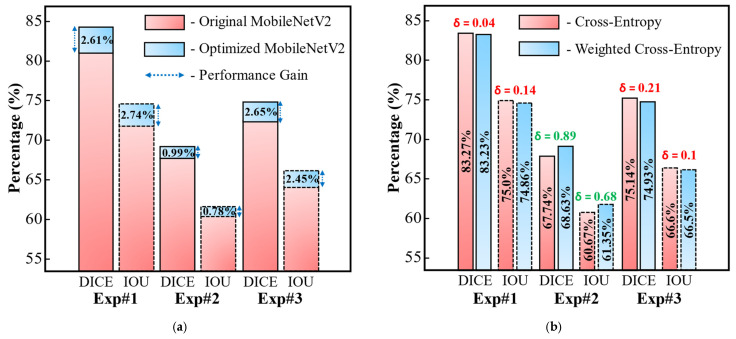
Quantitative results as ablation study to highlight the performance difference of (**a**) our proposed versus original MobileNetV2 as a backbone network, (**b**) weighted cross-entropy loss vs. simple cross-entropy loss function.

In our next ablation experiment, we highlight the quantitative performance gain of the proposed backbone network over the original MobileNetV2, as shown in Figure 5a. Compared to the original MobileNetV2, our backbone network achieves average gains with DICE scores of 2.61%, 0.99%, and 2.65% and IOU scores of 2.74%, 0.78%, and 2.45% for COVID-19-CT-Seg (Exp#1), MosMed (Exp#2) and cross-dataset (Exp#3), respectively (Figure 5a). Subsequently, we evaluated the performance of the proposed network with simple cross-entropy and compared its performance with that of our selected weighted cross-entropy loss function, as shown in Figure 5b. Compared with the original cross-entropy loss, weighted cross-entropy gives an additional gain as a DICE score of 0.89% and IOU score of 0.68% for the MosMed data (Exp#2). These results show that the weighted cross-entropy loss shows better performance in the case of minor lesion regions. In the case of COVID-19-CT-Seg (Exp#1) and cross-dataset (Exp#3), the weighted cross-entropy shows a small decrease in average performance. However, the average results (Exp#1, 2, and 3) show the superior performance of our selected loss function compared to the conventional cross-entropy loss. 

In addition, we highlight the quantitative impact of A-Block 1 (generating multiscale high-level features in the encoder) and A-Block 2 (provides multi-scale residual connection to the decoder) in the proposed network. The quantitative results in Table 2 show that both building blocks (A-Blocks 1 and 2) work in close symbiosis and progressively improve the overall performance of the proposed network. In detail, the addition of these two blocks (A-Block 1 and A-Block 2) gives average gains as DICE scores of 7.26% [83.23%−75.97%], 5.74% [68.63%−62.89%], and 3.89% [74.93%−71.04%]; PPV of 7.79% [76.69%−68.9%], 4.25% [62.00%−57.75%], and 4.27% [69.34%−65.07%]; and IOU scores of 7.53% [74.86%−67.33%], 4.26% [61.35%−57.09%], and 3.49% [66.5%−63.01%] for COVID-19-CT-Seg (Exp#1), MosMed (Exp#2), and cross-dataset (Exp#3), respectively. Similarly, we also observed small gains as SPE scores of 0.99% [99.18%−98.19%], 0.43% [99.41%−98.98%], and 0.4% [99.49%−99.09%] for COVID-19-CT-Seg (Exp#1), MosMed (Exp#2), and the cross-dataset (Exp#3), respectively. In addition to the superior performance gains of SPE, DICE, and IOU scores, a small decrease in the SEN value was noticed, particularly in the case of the COVID-19-CT-Seg data (Exp#1). However, the higher scores for the SPE, DICE, and IOU metrics show the superior aspects of these two building blocks (A-Block 1 and A-Block 2) in our network design.

Besides these three experiments (Exp#1, 2, and 3), the performance of the proposed network was further assessed for a mixed-dataset (comprising both COVID-19-CT-Seg and MosMed datasets). In this experiment, we aimed to highlight the performance of our method for a large variability of data. Therefore, we combined both datasets and obtained a new set of data that comprises a total of 70 COVID-19-positive cases (including a total of 5569 voxel images). After combining both datasets, we used 80% (56/70) for training and the remaining 20% (14/70) for testing. Table 1 presents the five-fold cross-validation results of this experiment. With the mixed dataset, our model exhibits average results of 89.8%, 99.25%, 72.55%, 79.56%, 70.96%, and 98.06% for SEN, SPE, PPV, DICE, IOU, and AUC, respectively. It can be observed from Table 3 that the average performance of the mixed dataset is higher than that of MosMed (Exp#2) but lower than that of COVID-19-CT-Seg (Exp#1). To be specific, the performance of the mixed dataset is higher than that of Exp#2 with average gains as PPV of 10.55% [72.55%−62.00%], DICE score of 10.93% [79.56%−68.63%], and IOU score of 9.61% [70.96%−61.35%] and lower that that of Exp#1 with average reductions in PPV of 4.14% [76.69%−72.55%], DICE score of 3.67% [83.23%−79.56%], and IOU score of 3.9% [74.86%−70.96%]. Such performance differences are caused by the high intra-class variability of the mixed dataset.

### 3.3. Comparisons with the State-of-the-Art Methods

A detailed comparison of our proposed network with the state-of-the-art deep segmentation models is presented in Table 4. In this comparison study, we evaluated the performance of various well-known segmentation models, such as SegNet (VGG16) [44], SegNet (VGG19) [44], U-Net [45], FCN [46], DeepLabV3+(ResNet) [22], and DeepLabV3+(MobileNetV2) [29], with our selected datasets under the same experimental protocol. Based on the results given in Table 4, we find DeepLabV3+(ResNet) [22] as the second-best network, which has approximately three times more parameters than the proposed network (i.e., 20.61 M [22] >> 6.65 M [Proposed]). In addition to the reduced number of parameters, our model provides additional performance gain in comparison with the second-best model [22]. In detail, the average gains of the proposed network compared with [22] (in the case of the COVID-19-CT-Seg data) are equal to 3.82% [91.19%−87.37%], 0.25% [99.18%−98.93%], 2.26% [76.69%−74.43%], 1.59% [83.23%−81.64%], 2.93% [74.86%−71.93%], and 1.43% [98.84%−97.41%] for SEN, SPE, PPV, DICE, IOU, and AUC, respectively. For the MosMed data, our model achieved a gain of 3.82% [89.45%−85.63%] for SEN with a small decrease in other performance metrics, as shown in Table 4. In the case of the cross-dataset, the performance gains of our model (versus [22]) are equal to 6.28% [73.2%−66.92%], 0.01% [99.49%−99.48%], 1.03% [69.34%−68.31%], 1.52% [74.93%−73.41%], 1.29% [66.5%−65.21%], and 1.42% [95.51%−94.09%] in terms of SEN, SPE, PPV, DICE, IOU, and AUC, respectively. In conclusion, these comparative results (Table 4) highlight the superior performance of our model over all baseline models [22,29,44,45,46].

Moreover, there are some existing studies [12,13,14,41,47,48,49,50] that provide state-of-the-art benchmarks for our selected datasets. Therefore, we also compared the results of our methods with those of these methods [12,13,14,41,47,48,49,50], which are given in Table 5. First, Zhang et al. [12] proposed a new variant of C-GAN, called CoSinGAN, with the capability to be learned from a single image and synthesize high-quality CT images for efficient training of a segmentation model. Their proposed CoSinGAN shows an average DICE score of 61.5% and 71.3% (for the COVID-19-CT-Seg data) applying 3-D and 2-D U-Net models, respectively. For the cross-dataset (i.e., training with COVID-19-CT-Seg and testing with MosMed data), CoSinGAN reached an average DICE score of 44.9% and 47.4% with 3-D and 2-D U-Net models, respectively. Later, Fan et al. [13] presented a semi-supervised learning method that utilizes unlabeled data in performing efficient training of their proposed segmentation network, called Inf-Net. Their method showed average DICE scores of 63.38% and 56.39% for COVID-19-CT-Seg (Exp#1) and cross-dataset (Exp#3 (fold 1)), respectively. Later, Ma et al. [14] developed three benchmarks for detection of right lung, left lung, and COVID-19-related findings using COVID-19-CT-Seg and MosMed datasets. A 3-D nnU-Net model [48] was used in [14], which showed average DICE scores of 67.3% and 58.85% for COVID-19-CT-Seg (Exp#1) and cross-dataset (Exp#3 (fold 1)), respectively. In the case of the cross-dataset, Ma et al.’s [14] given benchmarks showed better results than [12]. Subsequently, in [41,47,49,50], different segmentation methods (i.e., Miniseg [41], GASNet [47], Label-Free [49], and DASC-Net [50]) were proposed to achieve state-of-the-art results for effective segmentation of COVID-19 lesions in chest CT scans.

Based on the results given in Table 5, we find the existing GASNet [47], Miniseg [41], and DASC-Net [50] as the second-best methods in case of COVID-19-CT-Seg (Exp#1), MosMed (Exp#2) and the cross dataset (Exp#3 (fold 1)), respectively. In detail, our method shows superior results to GASNet (second-best) [47], with the average gains as SEN of 6.59% [91.19%−84.6%] and DICE score of 6.53% [83.23%−76.7%] for the COVID-19-CT-Seg dataset (Exp#1). In case of the MosMed dataset (Exp#2), the average gains of the proposed versus Miniseg (second-best) [41] are 9.83% [89.45%−79.62%], 1.7% [99.41%−97.71%], and 3.79% [68.63%−64.84%] for SEN, SPE, and DICE, respectively. Finally, in case of the cross-dataset (Exp#3 (fold 1)), our method also outperforms the DASC-Net (second-best) [50] with average gains as SEN of 3.98% [76.42%−72.44%] and DICE score of 11.84% [72.5%−60.66%]. Moreover, in a t-test analysis (proposed vs. second-best methods), we obtained an average *p*-value less than 0.05 (to be specific, average *p*-value = 0.044) that implies the discriminative performance of our model against [41,47,50] with a 95% confidence score. In conclusion, these comparative results highlight the superiority of our method over all the existing methods [12,13,14,41,47,48,49,50] related to the segmentation of COVID-19 lesions using chest CT scans.

**Figure 6:**
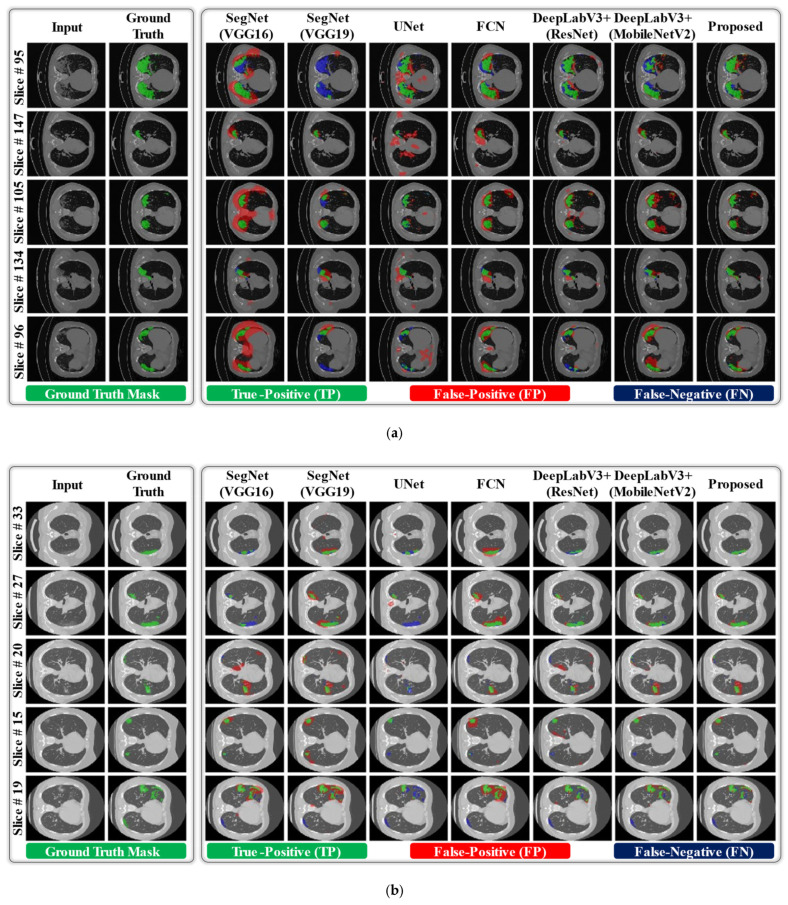
Visual comparison of segmentation results of the proposed DAL-Net with the other state-of-the-art models for infected data samples from the (**a**) COVID-19-CT-Seg data, (**b**) MosMed data.

**Figure 7:**
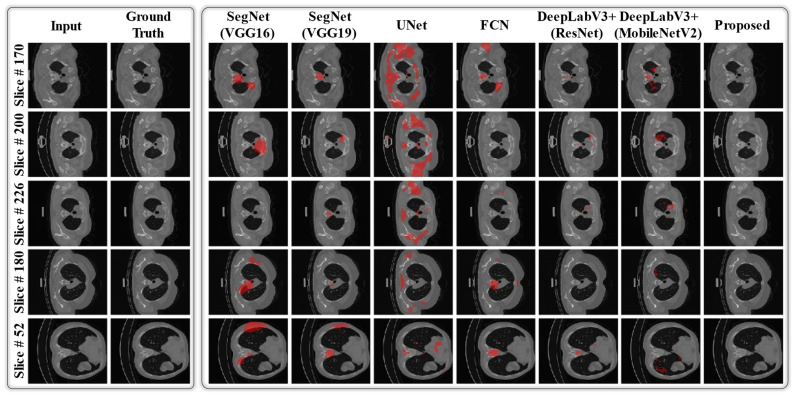
Visual comparison of segmentation results of the proposed DAL-Net with the other state-of-the-art models for normal data samples from the COVID-19-CT-Seg data. (“Red color: false-positive (FP)”).

Additionally, we visualized the lesion recognition results of the proposed model in comparison with the baseline models [22,29,44,45,46] for both datasets. In Figure 6, it can be observed that the lesion recognition results of the proposed network on both datasets are significantly related to the corresponding ground truths with a smaller number of FP and FN pixels in each sample image. In contrast, the various baseline models [22,29,44,45,46] provide inadequate results, showing numerous noninfected regions as FP pixels. In the case of small lesions (MosMed data), DeepLabV3+(ResNet) [22] and DeepLabV3+(MobileNetV2) [29] showed comparable results, as shown in Figure 6b. However, our method outperforms [22] and [29] based on the average quantitative results (as reported in Table 4). Such a visual comparison indicates the superior diagnostic performance of the proposed network over various state-of-the-art models. Moreover, Figure 7 presents the visual results of the proposed model in comparison with the baseline models for a few normal data samples (i.e., a CT image without including any lesion region). In Figure 7, it can be observed that our network successfully generates null output results and outperforms the baseline networks. Different baseline models segment out the normal pixels in a given CT image as lesion regions that are FP pixels (red color). In reality, the aggregation of multi-scale contextual features (output of A-Block 2 as a residual connection) with multi-scale high-level information (output of A-Block 1) resulted in the superior performance of the proposed network, particularly in the case of small lesions.

## 4. Discussion

This section discusses the principal findings of our method, including some limitations that may affect the performance of the system. Finally, we include a brief plan for our future work to overcome these limitations and further enhance the overall performance of the system.

### 4.1. Principal Findings

In the present disaster of COVID-19, CT scans are being considered as an effective diagnostic measure for the visual assessment of COVID-19-related findings, such as well-aerated regions, ground-glass opacity, crazy paving and linear opacities, and consolidation [3,4]. However, the visual assessment of CT scans is time-consuming process, particularly in case of trivial lesions. A recent breakthrough in deep learning models has boosted the diagnostic capability of CAD systems and further aided health professionals in making effective diagnostic decisions. In this study, we utilized the strength of recent deep learning methods to recognize the lesion regions related to COVID-19 infection using lung CT data. A novel deep segmentation model (namely DAL-Net) is mainly proposed, which includes a total of 6.65 million training parameters and efficiently identifies the infected regions (i.e., well-aerated regions, ground-glass opacity, crazy paving and linear opacities, and consolidation) in a given CT image. To be specific, the proposed method can be beneficial in the following aspects: 1) facilitating radiologists to identify trivial lesions in CT image, which may be overlooked due to human error; 2) providing an efficient way to quantify the proportion of the infected area of the lung; and 3) reducing the total diagnostic time of radiologists. In addition, the proposed framework can be used in the hospital setting by radiologists and clinicians in making an effective diagnostic decision. To address the generality issue, we considered two different datasets to develop and validate our proposed framework and further enhanced the performance of our network using data normalization technique (namely RH transformation). The experimental results (Table 1 and Figure 4) highlight the significant performance difference with and without applying data normalization in the case of cross-data analysis.

Our DAL-Net design aggregates multi-scale contextual features with high-frequency information using DL-convolution-based residual connectivity (A-Block 2), which improves the detection performance, particularly in the case of small lesion regions. The encoder module comprises basic structural units of MobileNetV2 along with a set of four multiscale DL-convolutional layers [22], which ultimately results in a faster execution speed and lower inference time. The average inference time of the proposed segmentation network was 43 ms on a single image, whereas the original MobileNetV2 takes approximately 51 ms. Owing to the optimal design of our backbone model, the average inference time of our backbone network was lower than that of the original MobileNetV2. The average inference time was calculated using the same computational environment as explained in Section 3.1. The fast execution speed and optimal memory consumption of our model make it applicable in real-time population screening applications based on visual data. Moreover, our model can analyze a huge collection of radiographic databases effectively and promptly, which makes it applicable in retrieval-based personalized diagnosis applications.

**Figure 8:**
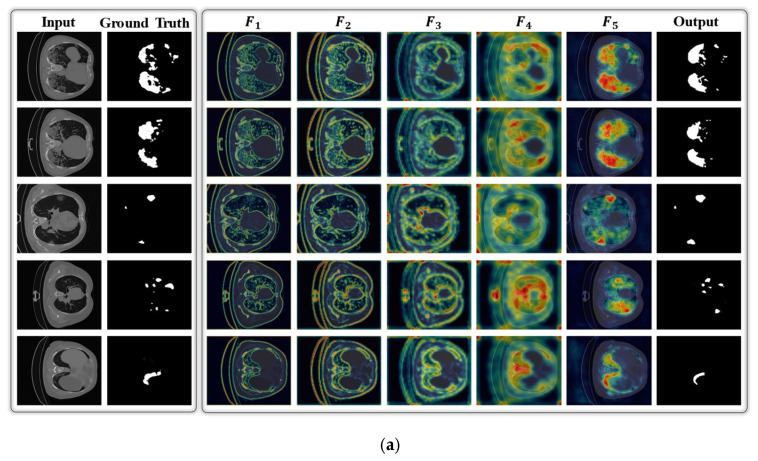

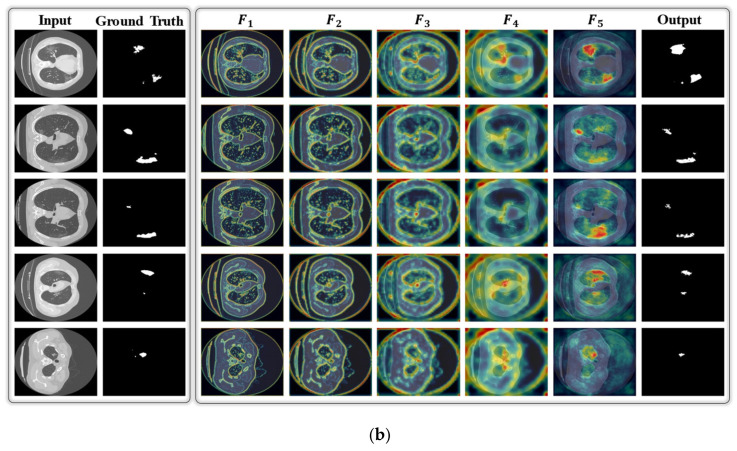
Visualization of multiple CAM outputs obtained from the different parts of the proposed DAL-Net for given data samples of the (**a**) COVID-19-CT-Seg data, (**b**) MosMed data.

In general, our deep segmentation network sequentially processes the given image through multiple layers and gradually activates the class-specific discriminative regions of COVID-19 infection as a class activation map (CAM) [51]. Figure 8 shows the successive activation of lesion regions (as CAM outputs) in the CT images. For each input image, five CAM outputs (labeled as $F_{1}$, $F_{2}$, $F_{3}$, $F_{4}$, and $F_{5}$ in Figure S1) were extracted from the five different layers of our network. Each CAM output is acquired by calculating the average response of all the extracted feature maps from a specific layer. In Figure 8, it can be observed that the lesion regions (in each input CT image) become more localized and distinctive after processing through successive layers of the network. Finally, we obtain a well-localized output as a binary image with a value of “1” (for infection class) and “0” (for normal/background class).

### 4.2. Limitations and Future Work

In spite of the superior performance of our method, there are still some limitations to the current study. Our selected datasets include only binary segmentation masks (either normal or diseased regions) as ground truth labels. Therefore, the multiclass infectious findings (i.e., lung sequelae, well-aerated regions, ground-glass opacity, crazy paving and linear opacities, and consolidation) related to COVID-19 are not distinguishable in this study. The proposed network can only differentiate between the normal and infectious regions in a given CT image. Additionally, some infectious findings (i.e., ground-glass opacity, consolidation) are not specific for COVID-19 [52]. Similar results can probably be found in case of influenza infection [52]. Therefore, the diagnostic performance of the proposed model can be degraded. However, additional RT-PCR and subjective assessment can be performed after obtaining positive results. After obtaining positive results, the accurate quantification of infected lung regions is essential for measuring infection severity in lung lobes and to find appropriate treatment [53]. In this regard, the proposed CAD solution can also assist the radiologists in quantifying the infected area of lung. In the future, we plan to collect more datasets including multiple diseases and develop a comprehensive framework that should be able to detect and differentiate multiple types of diseases, such as COVID-19 and other viral and bacterial infections. Additionally, we aim to enhance the overall diagnostic performance, particularly in the case of multi-source datasets. In spite of the superior results of our model over state-of-the-art methods, the cross-dataset performance is still limited. Therefore, we will include more diverse data in our future work and attempt to increase the generality of our method.

## 5. Conclusions

This paper presents an AI-driven CAD framework for the effective, timely, and well-localized recognition of COVID-19 infection in chest CT images. A lightweight deep segmentation network was developed and validated using two publicly available datasets. We mainly utilize the power of DW- and DL-convolution operations in our network design and proposed an optimal segmentation model including a total of 6.65 million parameters. The reduced size (to be specific, 9 MB) makes it easily applicable to mobile platforms to provide a fast evaluation of COVID-19-related lesions in chest CT images. In addition, a detailed cross-data analysis was performed to highlight the generality of the proposed model based on a real-world scenario. Our method shows average SEN of 91.19%, 89.45%, and 73.2%; SPE of 99.18%, 99.41%, and 99.49%; DICE scores of 83.23%, 68.63%, and 74.93%; and IOU scores of 74.86%, 61.35%, and 66.5% for COVID-19-CT-Seg, MosMed, and cross-dataset, respectively. Finally, a detailed comparative study further validated the superior performance (in terms of quantitative results and computational complexity) of the proposed model over various state-of-the-art methods. Our proposed model is publicly accessible for a fair comparison and further research and development.

## Figures and Tables

**Table 1:** All-fold cross-validation results of the proposed network for COVID-19-CT-Seg (Exp#1), MosMed (Exp#2), and cross-dataset (Exp#3 with and without RH transformation). The average scores are presented in boldface. (“Exp#: Experiment number”, “Avg.: Average”, “RH: Reinhard transformation”, “Std: Standard deviation”, “unit: %”).

Experiment#		#Fold	SEN	SPE	PPV	DICE	IOU	AUC
Same-Dataset	Exp#1 (COVID-19-CT-Seg)	1	87.75	99.02	81.22	86.26	78.21	98.83
		2	88.98	98.67	70.9	78.13	69.2	97.87
		3	93.01	99.06	74.57	81.93	73.22	99.06
		4	96.24	99.61	74.54	82.41	73.88	99.66
		5	89.97	99.54	82.2	87.42	79.8	98.79
		**Avg.** ± **Std**	**91.19** ± **3.43**	**99.18** ± **0.39**	**76.69** ± **4.84**	**83.23** ± **3.71**	**74.86** ± **4.22**	**98.84** ± **0.65**
	Exp#2 (MosMed)	1	87.76	99.2	59.22	65.05	58.59	97.55
		2	87.8	99.65	65.13	72.43	64.35	98.49
		3	93.98	99.43	60.79	67.42	60.36	99.06
		4	87.54	99.61	64.97	72.21	64.16	98.49
		5	90.15	99.17	59.9	66.03	59.27	98.74
		**Avg.** ± **Std**	**89.45** ± **2.75**	**99.41** ± **0.22**	**62.00** ± **2.84**	**68.63** ± **3.47**	**61.35** ± **2.73**	**98.47** ± **0.56**
Cross-Dataset	Exp#3 (Without RH)	1	71.88	99.63	63.7	69.76	62.18	95.43
		2	37.72	99.53	70.34	69.44	61.76	79.49
		**Avg.** ± **Std**	**54.8** ± **24.15**	**99.58** ± **0.07**	**67.02** ± **4.7**	**69.6** ± **0.23**	**61.97** ± **0.3**	**87.46** ± **11.27**
	Exp#3 (With RH)	1	76.42	99.69	66.01	72.5	64.41	96.09
		2	69.97	99.28	72.67	77.36	68.58	94.93
		**Avg.** ± **Std**	**73.2** ± **4.56**	**99.49** ± **0.29**	**69.34** ± **4.71**	**74.93** ± **3.44**	**66.5** ± **2.95**	**95.51** ± **0.82**

**Table 2:** Quantitative results highlight the significance of A-Block 1 and A-Block 2 in the proposed network. The best scores are presented in boldface. (“#Par.: Number of parameters”, “M: Million”, “Exp#: Experiment number”, “RH: Reinhard transformation”, “x: Not included”, “✓: Included”, “Std: Standard deviation”, “unit: %”).

Experiment#		A- Block 1	A- Block 2	#Par. (M)	SEN ± **Std**	SPE ± **Std**	PPV ± **Std**	DICE ± **Std**	IOU ± **Std**	AUC ± **Std**
Same-Dataset	Exp#1 (COVID-19-CT-Seg)	x	x	5.67	**92.49 ± ** **3.51**	98.19 ± 1.3	68.9 ± 5.26	75.97 ± 5.85	67.33 ± 5.37	98.62 ± 0.64
		x	✓	6.13	91.38 ± 4.56	98.99 ± 0.63	74.95 ± 6.86	81.49 ± 5.49	73.09 ± 5.95	98.5 ± 1.01
		✓	x	6.2	89.62 ± 2.58	98.6 ± 0.84	70.33 ± 5.35	77.25 ± 5.19	68.6 ± 5.02	98.11 ± 0.54
		✓	✓	6.65	91.19 ± 3.43	**99.18 ± ** **0.39**	**76.69 ± ** **4.84**	**83.23 ± ** **3.71**	**74.86 ± ** **4.22**	**98.84 ± ** **0.65**
	Exp#2 (MosMed)	x	x	5.67	89.7 ± 1.65	98.98 ± 0.39	57.75 ± 1.81	62.89 ± 2.71	57.09 ± 1.91	98.5 ± 0.24
		x	✓	6.13	**90.61 ± ** **4.39**	99.25 ± 0.4	61.02 ± 4.43	67.11 ± 5.51	60.29 ± 4.23	**98.6 ± ** **0.66**
		✓	x	6.2	90.14 ± 4.67	98.84 ± 0.51	57.32 ± 2.56	62.12 ± 3.76	56.58 ± 2.64	98.12 ± 0.75
		✓	✓	6.65	89.45 ± 2.75	**99.41 ± ** **0.22**	**62.00 ± ** **2.84**	**68.63 ± ** **3.47**	**61.35 ± ** **2.73**	98.47 ± 0.56
Cross-Dataset	Exp#3 (With RH)	x	x	5.67	72.05 ± 3.96	99.09 ± 0.86	65.07 ± 0.33	71.04 ± 0.17	63.01 ± 0.43	94.01 ± 2.04
		x	✓	6.13	**79.48 ± ** **8.3**	98.72 ± 1.44	64.88 ± 4.00	71.06 ± 3.95	63.0 ± 3.64	94.83 ± 2.23
		✓	x	6.2	76.95 ± 3.9	98.99 ± 0.72	63.3 ± 3.1	69.48 ± 3.54	61.82 ± 2.51	94.72 ± 2.45
		✓	✓	6.65	73.2 ± 4.56	**99.49 ± ** **0.29**	**69.34 ± ** **4.71**	**74.93 ± ** **3.44**	**66.5 ± ** **2.95**	**95.51 ± ** **0.82**

**Table 3:** All-fold cross-validation results of the proposed network for the mixed dataset (including both COVID-19-CT-Seg and MosMed datasets). The average scores are presented in boldface (“Exp#: Experiment number”, “Avg.: Average”, “Std: Standard deviation”, “unit: %”).

Experiment#		#Fold	SEN	SPE	PPV	DICE	IOU	AUC
Mixed-Datasets	Exp#1 (COVID-19-CT Seg) + Exp#2 (MosMed)	1	83.5	99.32	74.72	80.9	72.17	97.35
		2	88.28	98.63	65.86	73.02	64.53	96.89
		3	94.61	99.17	71.38	79.25	70.46	99.22
		4	95.56	99.6	72.21	80.23	71.56	99.24
		5	87.03	99.53	78.56	84.38	76.08	97.58
		**Avg. ± ** **Std**	**89.8 ± ** **5.15**	**99.25 ± ** **0.39**	**72.55** ± **4.67**	**79.56 ± ** **4.13**	**70.96 ± ** **4.17**	**98.06 ± ** **1.1**

**Table 4:** Performance comparisons of the proposed DAL-Net with the other state-of-the-art deep models. (“#Par.: Number of parameters”, “M: Million”, “Exp#: Experiment number”, “RH: Reinhard transformation”, “Std: Standard deviation”, “unit: %”).

**Experiment#**	**Models**	SEN ± **Std**	SPE ± **Std**	PPV ± **Std**	DICE ± **Std**	IOU ± **Std**	AUC ± **Std**
Exp#1 (COVID-19-CT-Seg)	SegNet (VGG16) [44]	**93.52** ± **4.49**	97.8 ± 1.63	66.58 ± 5.96	73.35 ± 6.78	64.97 ± 6.11	98.73 ± 0.69
	SegNet (VGG19) [44]	89.29 ± 5.56	98.45 ± 0.95	69.64 ± 6.52	76.31 ± 6.18	67.78 ± 5.97	98.55 ± 0.54
	U-Net (E.D 4) [45]	85.79 ± 7.04	93.76 ± 6.72	51.07 ± 15.82	73.78 ± 15.27	62.76 ± 8.38	96.83 ± 1.25
	FCN (USF 32) [46]	91.91 ± 5.55	97.77 ± 1.25	65.51 ± 4.74	72.23 ± 5.27	63.85 ± 4.63	98.41 ± 0.9
	DeepLabV3+(ResNet) [22]	87.37 ± 5.63	98.93 ± 0.89	74.43 ± 7.18	81.64 ± 5.73	71.93 ± 6.2	97.41 ± 1.3
	DeepLabV3+(MobileNetV2) [29]	90.62 ± 2.83	98.86 ± 0.79	73.88 ± 5.98	80.62 ± 5.63	72.12 ± 5.74	98.49 ± 0.97
	DAL-Net (Proposed)	91.19 ± 3.43	**99.18** ± **0.39**	**76.69** ± **4.84**	**83.23** ± **3.71**	**74.86** ± **4.22**	**98.84** ± **0.65**
Exp#2 (MosMed)	SegNet (VGG16) [44]	90.12 ± 2.62	97.99 ± 0.71	54.2 ± 0.79	57.17 ± 1.5	53.15 ± 1.11	98.35 ± 0.47
	SegNet (VGG19) [44]	**92.32** ± **2.84**	98.36 ± 0.61	55.19 ± 0.99	58.9 ± 1.76	54.32 ± 1.27	**98.78** ± **0.3**
	U-Net (E.D 4) [45]	89.67 ± 4.76	96.79 ± 1.9	53.18 ± 1.63	55.05 ± 3.24	51.54 ± 2.46	98.27 ± 0.43
	FCN (USF 32) [46]	89.94 ± 2.66	98.7 ± 0.39	56.15 ± 1.18	60.46 ± 1.88	55.41 ± 1.3	98.13 ± 0.42
	DeepLabV3+(ResNet) [22]	85.63 ± 3.1	**99.45** ± **0.23**	**62.43** ± **2.84**	**68.98** ± **3.42**	**61.62** ± **2.66**	97.7 ± 0.78
	DeepLabV3+(MobileNetV2) [29]	88.28 ± 2.81	99.37 ± 0.24	61.24 ± 2.44	67.64 ± 3.11	60.57 ± 2.37	98.15 ± 0.9
	DAL-Net (Proposed)	89.45 ± 2.75	99.41 ± 0.22	62.00 ± 2.84	68.63 ± 3.47	61.35 ± 2.73	98.47 ± 0.56
Exp#3 (Cross-Dataset (with RH))	SegNet (VGG16) [44]	72.65 ± 13.41	97.28 ± 3.22	58.53 ± 3.39	62.81 ± 4.72	56.47 ± 4.23	94.43 ± 0.93
	SegNet (VGG19) [44]	66.82 ± 14.8	99.02 ± 0.44	61.26 ± 3.14	66.24 ± 2.46	59.35 ± 1.56	95.1 ± 2.57
	U-Net (E.D 4) [45]	55.07 ± 21.99	97.88 ± 2.64	58.92 ± 3.42	62.22 ± 3.03	56.22 ± 2.9	91.96 ± 1.26
	FCN (USF 32) [46]	**78.15** ± **8.28**	98.73 ± 1.05	61.82 ± 1.89	67.86 ± 2.43	60.48 ± 1.47	95.01 ± 2.36
	DeepLabV3+(ResNet) [22]	66.92 ± 3.1	99.48 ± 0.32	68.31 ± 5.19	73.41 ± 4.68	65.21 ± 3.88	94.09 ± 0.08
	DeepLabV3+(MobileNetV2) [29]	73.26 ± 1.48	99.17 ± 0.79	66.2 ± 0.79	72.28 ± 0.79	64.05 ± 0.92	95.15 ± 0.71
	DAL-Net (Proposed)	73.2 ± 4.56	**99.49** ± **0.29**	**69.34** ± **4.71**	**74.93** ± **3.44**	**66.5** ± **2.95**	**95.51** ± **0.82**

**Table 5:** Performance comparisons of the proposed DAL-Net with the state-of-the-art methods related to the segmentation of COVID-19 lesions in chest CT scans. (“–: Not available”, “unit: %”).

Experiment#	Models	SEN	SPE	PPV	DICE	IOU	AUC
Exp#1 (COVID-19-CT-Seg)	CoSinGAN+3D U-Net [12]	**–**	**–**	**–**	61.5	**–**	**–**
	Inf-Net [13]	69.46	99.02	**–**	63.38	64.62	**–**
	3D nnU-Net [14,48]	**–**	**–**	**–**	67.3	**–**	**–**
	Label-Free [49]	66.2	**–**	**–**	69.8	**–**	**–**
	CoSinGAN+2D U-Net [12]	**–**	**–**	**–**	71.3	**–**	**–**
	Miniseg [41]	85.06	99.05	**–**	76.27	**84.49**	**–**
	GASNet [47]	84.6	**99.2**	**–**	76.7	**–**	**–**
	DAL-Net (Proposed)	**91.19**	99.18	**76.69**	**83.23**	74.86	**98.84**
Exp#2 (MosMed)	Inf-Net [13]	62.93	93.45	**–**	56.39	74.32	**–**
	Miniseg [41]	79.62	97.71	**–**	64.84	**78.33**	**–**
	DAL-Net (Proposed)	**89.45**	**99.41**	**62.00**	**68.63**	61.35	**98.47**
Exp#3 (Fold 1) (Cross-Dataset)	CoSinGAN+3D U-Net [12]	**–**	**–**	**–**	44.9	**–**	**–**
	CoSinGAN+2D U-Net [12]	**–**	**–**	**–**	47.4	**–**	**–**
	3D nnU-Net [14,48]	**–**	**–**	**–**	58.8	**–**	**–**
	GASNet [47]	60.4	**99.8**	**–**	58.9	**–**	**–**
	AFD-DA [50]	75.17	99.74	**–**	59.04	**–**	**–**
	DASC-Net [50]	72.44	99.78	**–**	60.66	**–**	**–**
	DAL-Net (Proposed)	**76.42**	99.69	**66.01**	**72.5**	**64.41**	**96.09**

## Data Availability

Not applicable.

## Supplementary Materials

The following are available online at https://www.mdpi.com/article/10.3390/jpm11101008/s1, Figure S1. Overall architecture of the proposed DAL-Net with preprocessing method, and Table S1. Layer-wise configuration details of the proposed DAL-Net.
