# Supplementary material for: Domain-Adaptive Artificial Intelligence-Based Model for Personalized Diagnosis of Trivial Lesions Related to COVID-19 in Chest Computed Tomography Scans
Source: J Pers Med. 2021 Oct 7;11(10):1008. doi: 10.3390/jpm11101008 (PMC8537687; doi:10.3390/jpm11101008)
Supplement: Supplementary file 1 [file jpm-11-01008-s001.zip › jpm-1342978-supplementary.pdf]

Journal of *Personalized Medicine*

Supplementary Materials

Domain-Adaptive Artificial Intelligence-Based Model for Personalized Diagnosis of Trivial Lesions Related to COVID-19 in Chest Computed Tomography Scans

Muhammad Owais, Na Rae Baek and Kang Ryoung Park *

Division of Electronics and Electrical Engineering, Dongguk University, 30 Pildong-ro 1-gil, Jung-gu, Seoul 04620, Korea; malikowais266@gmail.com (M.O.); naris27@dgu.ac.kr (N.R.B.)
* Correspondence: parkgr@dgu.edu; Tel.: +82-10-3111-7022

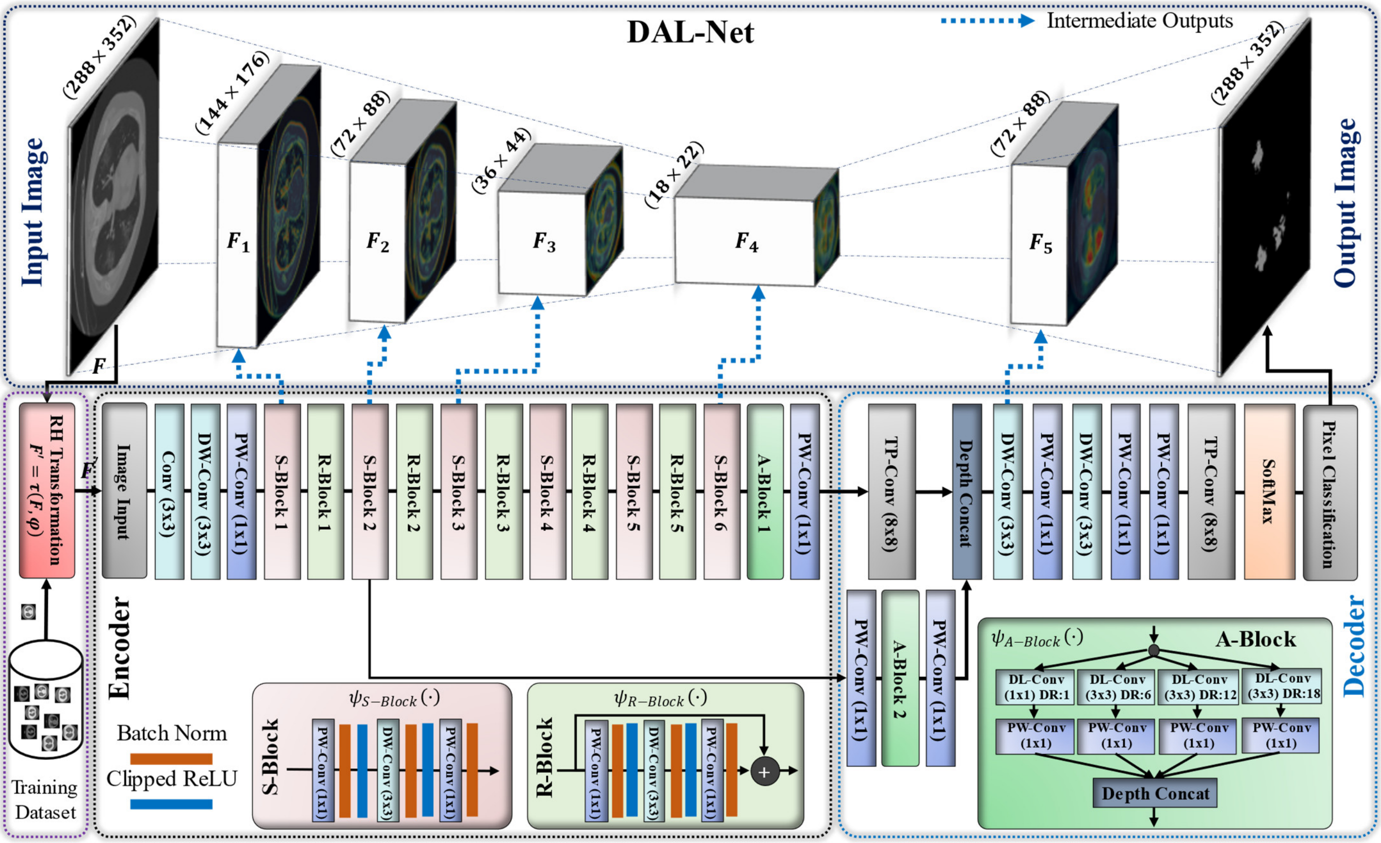

Figure S1. Overall architecture of the proposed DAL-Net with preprocessing method. ("Conv: Convolutional", "Depth Concat: Depth concatenation", "Crop Ref: Crop reference")

Table S1. Layer-wise configuration details of the proposed DAL-Net. ("$x^2 = x \times x$", "–: Not applicable", "#Par.: Number of parameters")

	Layer Name	Input Size	Output Size	Filter Size	#Filter	Stride	#Par.
Encoder	Image Input	288×352×3	–	–	–	–	–
	Conv	288×352×3	144×176×32	3^2	32	2	960
	DW-Conv	144×176×32	144×176×32	3^2	32	1	384
	PW-Conv	144×176×32	144×176×16	1^2	16	1	560
	S-Block 1	144×176×16	72×88×24	$1^2, 3^2, 1^2$	96,96,24	1,2,1	5,352
	R-Block 1	72×88×24	72×88×24	$1^2, 3^2, 1^2$	144,144,24	1,1,1	9,144
	S-Block 2	72×88×24	36×44×32	$1^2, 3^2, 1^2$	144,144,32	1,2,1	10,320
	R-Block 2	36×44×32	36×44×32	$1^2, 3^2, 1^2$	192,192,32	1,1,1	15,264
	S-Block 3	36×44×32	18×22×64	$1^2, 3^2, 1^2$	192,192,64	1,2,1	21,504
	R-Block 3	18×22×64	18×22×64	$1^2, 3^2, 1^2$	384,384,64	1,1,1	55,104
	S-Block 4	18×22×64	18×22×96	$1^2, 3^2, 1^2$	384,384,96	1,1,1	67,488
	R-Block 4	18×22×96	18×22×96	$1^2, 3^2, 1^2$	576,576,96	1,1,1	119,520
	S-Block 5	18×22×96	18×22×160	$1^2, 3^2, 1^2$	576,576,160	1,1,1	156,576
	R-Block 5	18×22×160	18×22×160	$1^2, 3^2, 1^2$	960,960,160	1,1,1	322,080
	S-Block 6	18×22×160	18×22×320	$1^2, 3^2, 1^2$	960,960,320	1,1,1	476,160
	A-Block 1	18×22×320	18×22×1024	$1^2, 3^2, 3^2, 3^2$ (1,6,12,18)* $1^2, 1^2, 1^2, 1^2$	320,320,320,320 256,256,256,256	1,1,1,1 1,1,1,1	340,992
	PW-Conv	18×22×1024	18×22×256	1^2	256	1	262,912
Decoder	TP-Conv	18×22×256	72×88×256**	8^2	256	4	4,194,560
	PW-Conv	72×88×144	72×88×320	1^2	320	1	47,040
	A-Block 2	72×88×320	72×88×1024	$1^2, 3^2, 3^2, 3^2$ (1,6,12,18)* $1^2, 1^2, 1^2, 1^2$	320,320,320,320 256,256,256,256	1,1,1,1 1,1,1,1	340,992
	PW-Conv	72×88×1024	72×88×48**	1^2	48	1	49,296
	Depth Concat	**	72×88×304	–	–	–	–
	DW-Conv	72×88×304	72×88×304	3^2	304	1	3,040
	PW-Conv	72×88×304	72×88×256	1^2	256	1	78,592
	DW-Conv	72×88×256	72×88×256	3^2	256	1	2,560
	PW-Conv	72×88×256	72×88×256	1^2	256	1	66,304
	PW-Conv	72×88×256	72×88×2	1^2	2	1	514
	TP-Conv	72×88×2	288×352×2	8^2	2	4	258
	SoftMax	288×352×2	288×352×2	–	–	–	–
	Pixel Classification	–	–	–	–	–	–
**Total learning parameters: 6,647,476**							

* Dilation factor, **Input feature vectors fed to depth concatenation (Depth Concat) layer.
